# Disseminated intravascular coagulation: cause, molecular mechanism, diagnosis, and therapy

**DOI:** 10.1002/mco2.70058

**Published:** 2025-01-14

**Authors:** Fangchen Gong, Xiangtao Zheng, Shanzhi Zhao, Huan Liu, Erzhen Chen, Rongli Xie, Ranran Li, Ying Chen

**Affiliations:** ^1^ Department of Emergency Ruijin Hospital, Shanghai Jiao Tong University School of Medicine Shanghai China; ^2^ Shanghai Institute of Aviation Medicine, Shanghai Jiao Tong University Medical School Affiliated Ruijin Hospital Shanghai China; ^3^ Department of General Surgery Ruijin Hospital Lu Wan Branch, Shanghai Jiaotong University School of Medicine Shanghai China; ^4^ Department of Critical Care Medicine Ruijin Hospital, Shanghai Jiao Tong University School of Medicine Shanghai China; ^5^ Department of Emergency and Critical Care Medicine Ruijin Hospital Wuxi Branch, Shanghai Jiao Tong University School of Medicine Wuxi China

**Keywords:** coagulation, disseminated intravascular coagulation, fibrinolysis, sepsis, trauma

## Abstract

Disseminated intravascular coagulation (DIC) is a complex and serious condition characterized by widespread activation of the coagulation cascade, resulting in both thrombosis and bleeding. This review aims to provide a comprehensive overview of DIC, emphasizing its clinical significance and the need for improved management strategies. We explore the primary causes of DIC, including sepsis, trauma, malignancies, and obstetric complications, which trigger an overactive coagulation response. At the molecular level, DIC is marked by excessive thrombin generation, leading to platelet and fibrinogen activation while simultaneously depleting clotting factors, creating a paradoxical bleeding tendency. Diagnosing DIC is challenging and relies on a combination of existing diagnostic criteria and laboratory tests. Treatment strategies focus on addressing the underlying causes and may involve supportive care, anticoagulation therapy, and other supportive measures. Recent advances in understanding the pathophysiology of DIC are paving the way for more targeted therapeutic approaches. This review highlights the critical need for ongoing research to enhance diagnostic accuracy and treatment efficacy, ultimately improving patient outcomes in those affected by DIC.

## Graphical abstract

DIC course: imbalance of coagulative system, anticoagulant activity, and fibrinolysis system. DIC includes two phenotypes: thrombotic and fibrinolytic. The thrombotic phenotype includes coagulation activation, insufficient anticoagulation, and hypofibrinolysis, indicating a proclivity for clot formation. Conversely, the fibrinolytic phenotype is characterized by hyperfibrinolysis, which suggests an increased risk of bleeding due to excessive clot breakdown

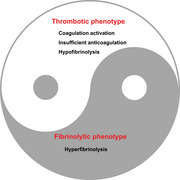

## 1 INTRODUCTION

Disseminated intravascular coagulation (DIC) is an acquired syndrome characterized by widespread microvascular thrombosis and simultaneous consumption of platelets and clotting factors. This leads to multiple organ dysfunction and uncontrolled bleeding, contributing significantly to morbidity and mortality in sepsis and other critical illnesses.^1^ DIC imposes a substantial economic burden, with patients experiencing higher mortality rates, prolonged hospital stays, and increased medical costs. These factors underscore the urgent need for effective diagnostic and treatment strategies to alleviate the human and financial toll of DIC.^2^, ^3^

The clinical importance of DIC lies in its potential to cause widespread organ damage due to microvascular thrombosis and subsequent hemorrhage due to consumptive coagulopathy and fibrinolysis. The scope of this review encompasses the pathophysiological mechanisms of DIC, with a particular focus on the role of microthrombi in the context of underlying diseases.

Furthermore, we will delve into the molecular mechanisms of DIC, highlighting the interplay between the immune system and coagulation, anticoagulant activity, the end‐stage consumption of coagulant factors, and fibrinolysis system. The activation of coagulative disorders is secondary to many clinical conditions, and timely diagnosis and treatment of DIC are vital to prevent disease progression and reduce mortality. We will explore the current diagnostic criteria for DIC and the challenges they present in the early identification of the hypercoagulable state, which is crucial for initiating prompt and effective treatment.

The review also addresses the challenges of current treatment strategies for DIC, which vary greatly depending on the underlying causative diseases. There is a pressing need for clinicians to better understand the importance of DIC, especially in recognizing the early signs of the hypercoagulable state and initiating appropriate therapeutic interventions. By providing a comprehensive overview of DIC, this review aims to guide clinical management and improve outcomes for patients affected by this potentially devastating syndrome.

## 2 EPIDEMIOLOGY, ETIOLOGY, AND CAUSES OF DIC

Elucidating the epidemiology of DIC should not ignore its heterology. DIC is a complication of many diseases, such as severe systemic infections, trauma, malignant tumors, vascular malformations, severe immune reactions, heatstroke, and so on (Table 1).

**TABLE 1:** DIC causes.

Etiology category	Causes	Estimated incidence	Prognosis
Infectious diseases	Sepsis, bacterial pneumonia	30–50%	Mortality rate 40%; timely antibiotic treatment and supportive care are crucial.
Trauma	Severe injury, traffic accidents	10–50% head trauma 36–41%	Mortality rate 25–34%; related to the severity of trauma and timely medical intervention; may progress to multiple organ failure
Solid tumors^15^	Pancreatic, gastric, lung cancer	5–15%	Related to cancer type, stage, and treatment. Compared with patients without DIC, those with early and late‐stage malignant tumors who developed DIC had lower survival rates.
Hematological cancers^16^, ^17^, ^18^	ALL	15–20%	
	APL	70–80%	Mortality rate 20% (30 days)
	AML	20%	Mortality rate 42.5% (30 days)
Obstetric complications^19^	Abruptio placentae, severe preeclampsia	1% 0.2%	Mortality rate 1%; emergency situations requiring rapid diagnosis and management; prognosis is related to maternal and fetal conditions.
Heat stroke^20^, ^21^, ^22^	Heat stroke due to high temperatures	9.6–28.4%	Mortality rate 26%
Snake bite^23^, ^24^	Snake bite	25–50%, geographically dependent	
Out‐of‐hospital cardiac arrest^25^		10–30%	Mortality rate 83%
Vascular abnormality		A small percentage	
Immune‐mediated diseases	Systemic lupus erythematosus, antiphospholipid syndrome	Small proportion	Related to disease control and immunosuppressive therapy; may affect long‐term prognosis

Abbreviations: ALL, acute lymphoblastic leukemia; AML, acute myeloid leukemia; APL, acute promyelocytic leukemia.

Although the clinical manifestations are diverse, essentially, DIC is caused by coagulative disorders imbalanced by endogenous anticoagulant and fibrinolytic mechanisms. Excessive activation of thrombin leads to proteolytic conversion of fibrinogen and the formation of fibrin within the vasculature. If the consumption of coagulation factors exceeds the hepatic output of synthesis, consumptive coagulopathy occurs, accompanied by thrombocytopenia, which signals an increased risk of bleeding. Statistics on the incidence and outcome of DIC varied because patients with this condition often have underlying diseases and additional causes, leading to diagnostic delay and inaccuracy.^4^ Besides, different DIC score has been established, this may lead to statistical differences in the disease across different countries and hospitals, depending on the score system that has been employed. The differences and linkage between different score system will be discussed in the diagnosis section.

The incidence of DIC ranges from 18 to 32% in intensive care unit (ICU) patients based on ISTH diagnostic criteria, while the incidence is 8.5% base on JAAM DIC.^4^, ^5^, ^6^, ^7^ The 28‐day mortality rate for DIC is about 20–50%. Sepsis and septic shock can lead to the occurrence of DIC due to factors such as the cytokine storm and endothelial injury, while the progression of DIC can further exacerbate organ dysfunction. Sepsis is the most common cause of DIC. The incidence of DIC in patients with sepsis is high as 46.8% based on JAAM DIC.^2^ The prevalence ranges from 56.1 to 60.8% base on ISTH SIC criteria.^3^, ^8^ In Europe, SIC prevalence was 22.1% according to the HYPRESS trial, in which sepsis diagnosis based on SEPSIS‐3 was evaluated.^9^ The mortality of sepsis‐associated DIC amounts to 30%, irrespective of the diagnostic standards used. Trauma, particularly severe injury associated with substantial tissue damage and shock, can account for a considerable percentage of DIC cases. Patients with head trauma could have incidence of DIC amount to 30–40%.^10^, ^11^ Approximately 7% of individuals with solid malignancies exhibit DIC, a figure that escalates with the progression of the disease and in those considered at risk for thrombotic events.^12^ Furthermore, DIC is identified in a considerable proportion of patients afflicted with hematological malignancies, with a heightened incidence in cases of acute leukemia (15–17%).^13^ Complications such as abruptio placentae, amniotic fluid embolism, and eclampsia are significant causes of DIC in pregnant women.^14^ Other causes of DIC are associated with vascular abnormalities, liver diseases, immune reactions, toxins, and transfusion reactions, all having their instinct pathophysiology.

The prognosis of DIC is closely tied to the underlying cause and the speed of diagnosis and treatment. Mortality rates are high, particularly in cases driven by sepsis, cancer, or major trauma. Early recognition and treatment of the underlying disorder, along with supportive care, can improve outcomes (Table 1).

## 3 PATHOPHYSIOLOGY

### 3.1 Waterfall theory and the cell‐based theory

DIC is characterized by a disruption in the balance between coagulation and bleeding within the body. To understand its pathophysiological mechanisms, two theories are pivotal: the waterfall theory and the cell‐based theory.

In the early 1960s, MacFarlane, Davie, and Ratnoff put forward the “waterfall theory” of coagulation.^26^, ^27^ They proposed that blood coagulation factors exist in an inactive proenzyme form, and the activation of one factor triggers a sequence of proteolytic reactions. This cascade activates subsequent enzymes, leading to the formation of thrombin, which cleaves fibrinogen to form a fibrin clot, causing blood to coagulate. Despite its wide recognition, this theory cannot explain certain phenomena, such as the lack of bleeding in patients with deficiencies in coagulation factor XII (FXII), prekallikrein (PK), and high‐molecular‐weight kininogen, despite their prolonged activated partial thromboplastin time (APTT).^28^ Hemophilia patients with deficiencies in FVIII and FIX also have a significantly prolonged APTT, but show a clear bleeding tendency.

**FIGURE 1:**
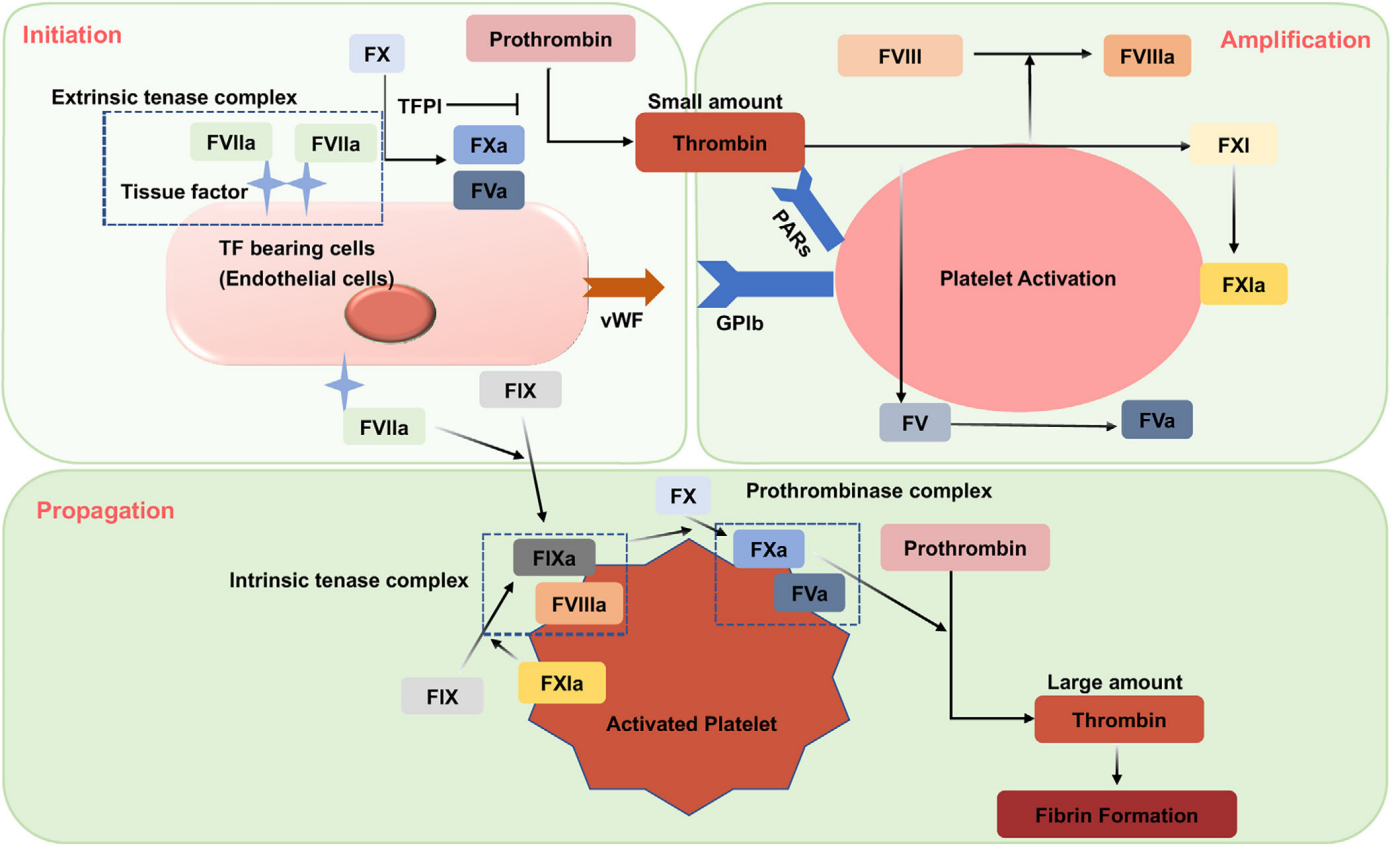
Cell‐based model of coagulation. The cell‐based model of DIC emphasizes the role of cellular interactions in coagulation. Initiation occurs on TF‐bearing cells via the extrinsic pathway, leading to thrombin generation. This thrombin activates more coagulation factors and platelets, amplifying the response. The propagation phase, primarily on platelet surfaces, generates a large burst of thrombin necessary for fibrin clot formation, a key step in the coagulation process.

The cell‐based hemostasis model that has developed in the past decade can better explain the hemostasis process and is gradually being accepted^29^ (Figure 1). It proposed that coagulation occurs not as a cascade, but is regulated by properties of cell surfaces. The process is divided into three overlapping steps: initiation, amplification, and propagation.^30^

During the initiation phase, various factors, such as endothelial injury or inflammatory stimuli induced tissue factor (TF) expression on TF bearing cells. Coagulation commences when TF binds to factor VII, forming the extrinsic tenase complex. This complex activates FIX and FX at the site of injury.^31^ FXa is quickly neutralized by TF pathway inhibitor (TFPI), resulting in a small amount of thrombin production. The thrombin spark emerges as the central event in the amplification and propagation phase. Thrombin activates platelets via protease‐activated receptors (PARs), notably PAR‐4.^32^ This activation leads to the exposure of procoagulant phospholipids, such as phosphatidylserine and phosphatidylethanolamine, on the platelet surface to activate coagulation factors.^33^ This activation results in the exposure of procoagulant phospholipids, like phosphatidylserine and phosphatidylethanolamine, on the platelet surface, which further activates coagulation factors.^34^ Thrombin also activates FXI, FVIII, and FV by binding to the GP1b receptors on the platelet surface, thus initiating the amplification phase. FXIa amplifies the conversion of FIX to FIXa by the extrinsic tenase. Thrombin interacts with PAR‐1 on endothelial cells, activating inflammatory signaling pathways that lead to the release of von Willebrand factor (vWF), angiopoietin‐2, and P‐Selectin, thereby amplifying the inflammatory response and inducing microthrombus formation.^35^ During the propagation phase, FVIIIa and FIXa form the intrinsic tenase complex, generating a substantial amount of FXa. FXa with FVa forms the prothrombinase complex, which converts prothrombin to thrombin in large quantities.^36^ Thrombin also transforms fibrinogen into fibrin, cross‐links platelets via the GPIIb/IIIa receptor, creating a stable clot. Platelet provide a reaction platform to generate a large amount of thrombin, which in turn recruits and activates more platelets, so thrombin activating platelets is an important step in coagulation.

Coagulation termination is regulated by physiological anticoagulant pathways, including antithrombin (AT), activated protein C (APC), and TFPI. Fibrin within blood vessels triggers the plasmin‐mediated breakdown of fibrin itself. The fibrinolytic system, which is central to this process, is primarily composed of plasmin, plasminogen activators (PAs), and plasminogen activator inhibitors (PAIs).^37^

Together, the waterfall theory and the cell‐based theory provide a more complete understanding of the complex processes involved in DIC. The waterfall theory provides a foundational framework for the sequence of coagulation events, while the cell‐based theory adds depth by accounting for the cellular interactions and regulatory mechanisms that are crucial for maintaining hemostasis and preventing pathological thrombosis. Ultimately, DIC presents a prothrombotic state marked by thrombin‐induced platelet activation, intensification of the coagulation sequence, and fibrin clot formation. Yet, with the progression of DIC, the consumption of coagulation factors and platelets leads to a transition toward hypo‐coagulability, which in turn, increases the propensity for bleeding.

### 3.2 Phenotypes of DIC

**FIGURE 2:**
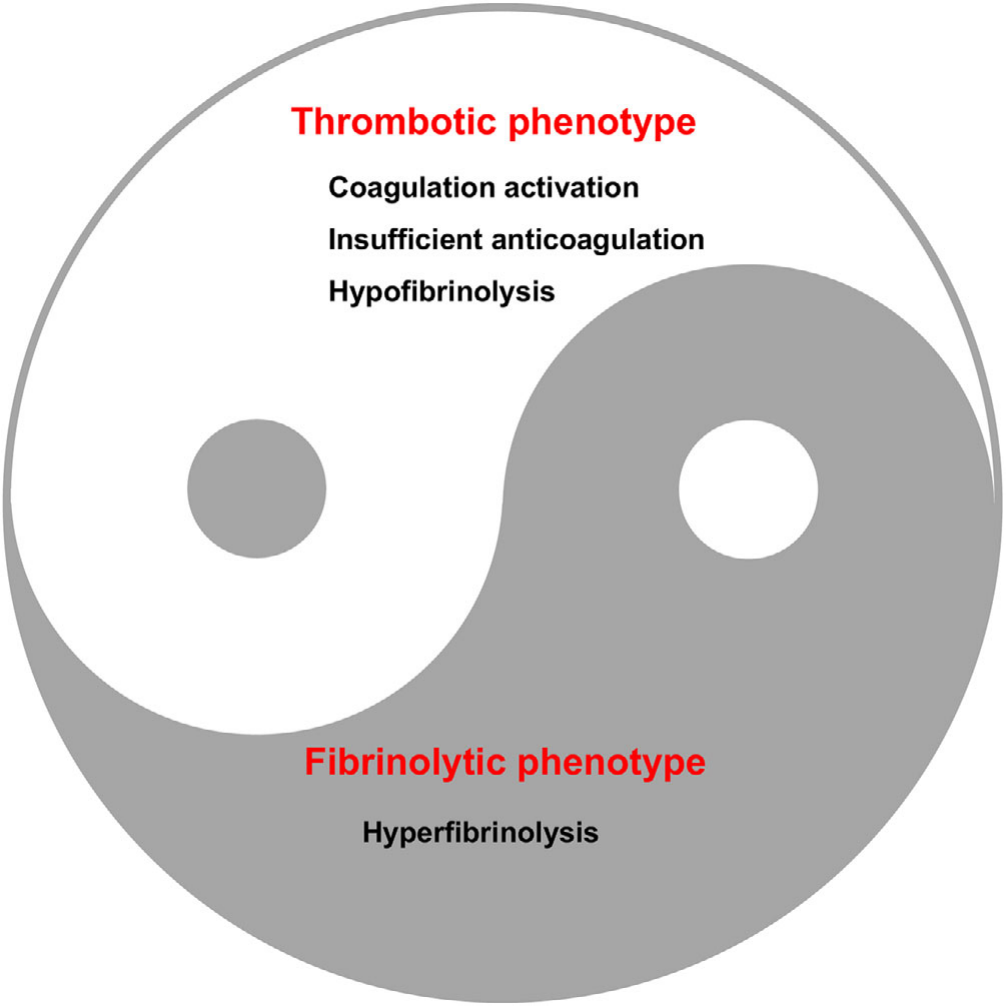
DIC course: imbalance of coagulative system, anticoagulant activity, and fibrinolysis system. Two phenotypes of DIC: thrombotic and fibrinolytic. The thrombotic phenotype includes coagulation activation, insufficient anticoagulation, and hypofibrinolysis, indicating a proclivity for clot formation. Conversely, the fibrinolytic phenotype is characterized by hyperfibrinolysis, which suggests an increased risk of bleeding due to excessive clot breakdown.

The essence of DIC pathophysiology lies in the disruption of the equilibrium between procoagulant and anticoagulant forces. This imbalance leads to an uneven consumption of various components, which is difficult to predict and fluctuates throughout the disease's progression, ultimately influencing the dominant clinical phenotype. DIC is classified into thrombotic and fibrinolytic phenotypes, based on the extent of thrombosis and hemorrhage, with each phenotype being characterized by thrombosis and bleeding, respectively^38^ (Figure 2).

Thrombotic DIC is marked by the activation of coagulation, insufficient anticoagulation, endothelial damage, and inhibition of fibrinolysis by PAI‐1. These factors lead to the formation of microvascular fibrin thrombi and subsequent organ dysfunction. Additionally, the overproduction of thrombin depletes platelets and coagulation factors, resulting in a condition known as consumption coagulopathy. This is evidenced by slow, seeping bleeding, often in mucosal areas, puncture sites of blood vessels, and regions of injury or surgical intervention.^37^ It is important to recognize that the thrombotic form of DIC is also associated with a certain level of consumptive hemorrhage.

DIC with a fibrinolytic phenotype is defined by excessive thrombin generation and systemic pathological hyperfibrinolysis due to the underlying disease, which leads to severe bleeding as a result of excessive plasmin formation.^39^ In sepsis‐related DIC, the typical pathological scenario is an excessive clotting process accompanied by a suppression of fibrinolysis, which results in thrombotic organ damage. In contrast, DIC linked to trauma is characterized by severe coagulation factors consumption coupled with sudden hyperfibrinolysis.^40^

It is of utmost importance to understand the molecular mechanisms that link physiological processes in DIC, as this knowledge is essential for developing effective diagnostic and therapeutic strategies.

## 4 MOLECULAR MECHANISM

### 4.1 Initiation of coagulative disorders

#### 4.1.1 Increased TF activity

The TF–FVIIa complex is the principal initiator of coagulation activation. TF, serving as a high‐affinity receptor and cofactor for factor FVII/VIIa, is ubiquitously expressed on various cell types, including monocytes, endothelial cells, platelets, lymphocytes, and cancer cells.^41^, ^42^, ^43^ During acute inflammation or sepsis, pattern recognition receptors (PRRs) bind to both pathogen‐associated molecular patterns (PAMPs) and damage‐associated molecular patterns (DAMPs), inducing TF expression primarily on monocytes and initiating procoagulant responses.^44^, ^45^ Activated monocytes also releases extracellular vesicles expressing TF and phosphatidylserine on their surfaces, activate the intrinsic and extrinsic coagulation pathways.^46^ These macrovesicles, rich in TF, can attach to activated platelets, neutrophils, and endothelial cells. The inflammatory response triggered by PAMPs also induces TF expression on endothelial cells, further promoting the coagulation cascade.^47^, ^48^ Activated vascular endothelial cells, platelets, and extracellular vesicles amplify TF responses, accelerating the procoagulant state.^49^ Intracellular immune sensors, such as DAMP‐induced inflammasomes, also stimulate TF release through pyroptosis.^50^ In addition to coagulation initiation, TF also plays a crucial role in inflammation, serving as a link between the inflammatory and coagulation pathways. TF possesses signaling activity that promotes a range of inflammatory responses. This signaling occurs through PARs in conjunction with other coagulation factors, leading to the expression of proinflammatory cytokines and the modulation of endothelial phenotype.^51^ A recent study found that TF binds to the interferon‐alpha receptor 1 and antagonizes its signaling, preventing spontaneous sterile inflammation and maintaining immune homeostasis.^52^ In animal models of sepsis, the genetic deletion of TF or its inhibition with neutralizing antibodies can prevent coagulation initiation and reduce mortality rates.^53^, ^54^ However, due to TF's significant physiological role in sepsis, clinical research on systemic TF inhibitors is challenging. In the context of cancer, TF increases the clotting propensity of tumors and facilitates interactions between platelets and cancer cells, playing a crucial role in tumor dissemination through the bloodstream.^54^ Cancer‐derived TF vesicles play a pivotal role in coagulation. These vesicles activate the extrinsic coagulation cascade, leading to fibrin deposition and thrombus formation. Cancer‐derived TF vesicles are instrumental in activating the extrinsic coagulation cascade, leading to fibrin deposition and thrombus formation.^55^ Additionally, TF vesicles from noncancer cells and circulating tumor cells contribute to coagulation. Soluble TF, which lacks transmembrane and cytoplasmic domains, may also be associated with EVs on cancer cells, potentially enhancing procoagulant activity. Silencing TF within tumors can effectively curb metastasis and mitigate cancer‐associated hypercoagulability in mouse models.^56^

#### 4.1.2 Platelet

Platelet activation and aggregation are crucial in clot formation during DIC. Platelets are the first responders to damaged blood vessels, and underlying causes of DIC, such as endothelial damage, pathogen contact, or inflammatory factors, trigger platelet aggregation.^57^ Cell‐free DNA and histones, bacterial lipopolysaccharides (LPSs), and neutrophil extracellular traps (NETs) in sepsis can directly activate platelets. The activation of the coagulation cascade by TF and the subsequent production of thrombin promote not only fibrin formation but also strong platelet activation. Once activated, platelets provide more and higher‐affinity binding sites for activated coagulation factors.^58^ Activated platelets can further promote monocyte TF expression and fibrin formation by expressing P‐selectin and facilitate the adhesion of platelets to leukocytes and the vascular wall. Additionally, large amounts of vWF released due to inflammation‐induced endothelial damage contribute to increased platelet‐vessel wall interactions in DIC. vWF, an acute‐phase factor upregulated and released during systemic inflammation, enhances platelet adhesion and aggregation in the microcirculation.^59^ Patients with DIC exhibit higher platelet activation than those without DIC.^60^ While platelet count is considered a criterion in DIC diagnosis, examining platelet function is also vital for identifying patients at high risk of developing DIC.

### 4.2 Crosstalk between inflammation and DIC

**FIGURE 3:**
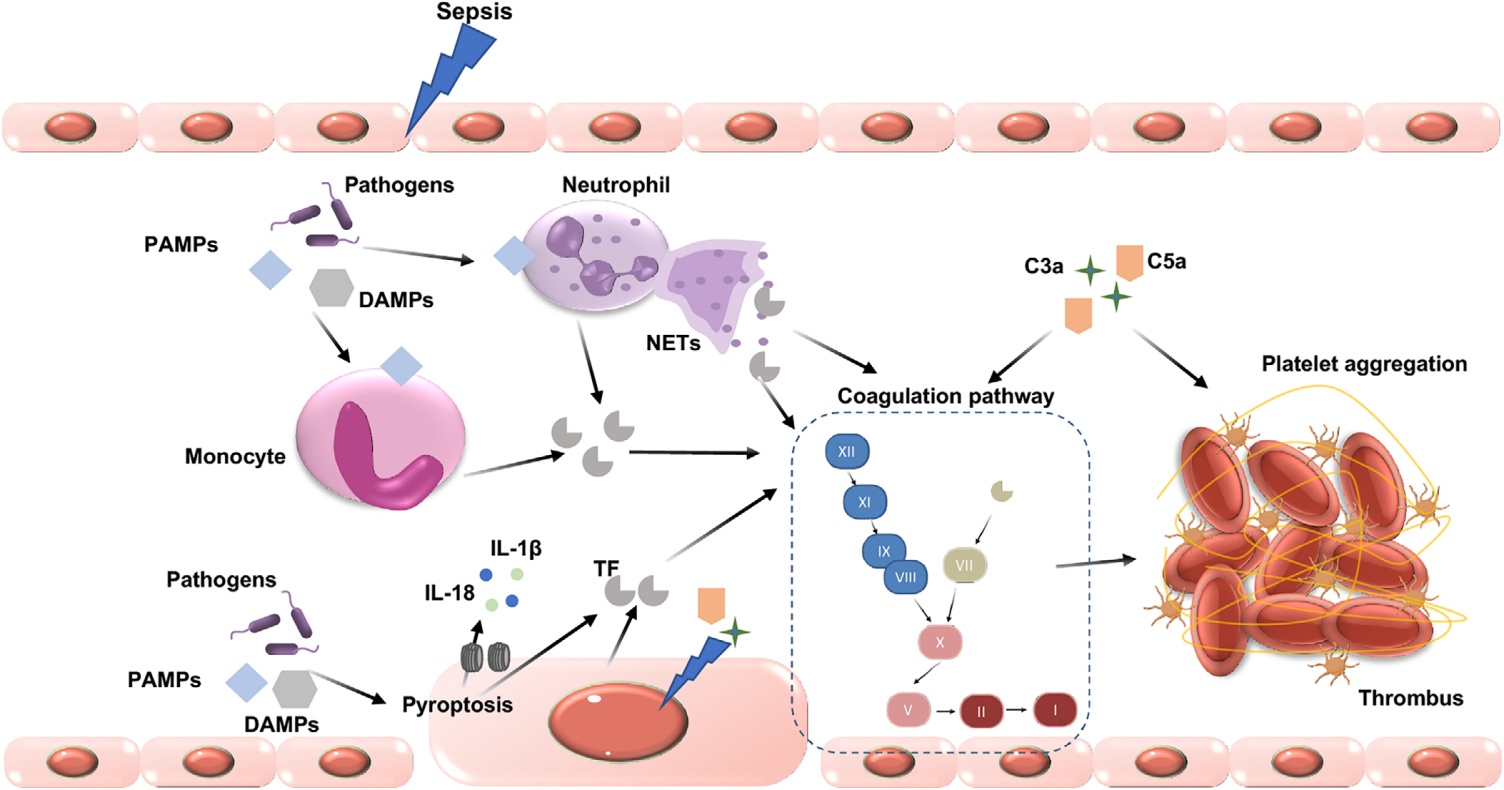
Crosstalk between inflammation and DIC. Schematic representation of the interplay between trauma, pathogen‐associated molecular patterns (PAMPs), damage‐associated molecular patterns (DAMPs), immune response, and coagulation pathways leading to thrombus formation. Trauma and pathogens trigger neutrophil activation, resulting in the release of NETs and monocyte activation. The activated monocytes express TF, further amplifying the coagulation cascade. Intracellular immune sensors, such as DAMP‐induced inflammasomes, also stimulate TF release through pyroptosis. Complement components C3a and C5a also contribute to platelet aggregation, which together with the coagulation cascade, leads to thrombus formation.

The relationship between the inflammatory response and DIC is complex, involving numerous molecular mechanisms (Figure 3). When the immune system encounters stimuli such as infections or trauma, it initiates a cascade that activates coagulation pathways. Monocytes and macrophages, equipped with PRRS like Toll‐like receptors, Fcγ‐receptors, and G‐protein‐coupled receptors, identify PAMPs. This detection process leads to the convergence of the innate immune response and the coagulation pathway. Proinflammatory cytokines, including tumor necrosis factor (TNF)‐α, interleukin‐1β (IL‐1β), and IL‐6, may act as procoagulants, although the exact mechanisms are not fully understood.^61^ For instance, TNF‐α exposure to whole blood leads to platelet aggregation and activation. However, TNF‐α can also decrease platelet activation by inhibiting thrombi formation through NO generation.^62^, ^63^ IL‐6 has been suggested to potentially play a role in the coagulation activation by inducing TF triggered by endotoxins. However, it seems that IL‐6 does not contribute to coagulation activation caused by LPSs in humans.^64^ In the body's complex environment, interactions between inflammatory cytokines and other molecules can produce varying coagulation effects.

#### 4.2.1 DAMPs and DIC

Growing evidence suggests that DAMPs, such as NETs, extracellular DNA, high mobility group box 1 protein (HMGB1), S100 proteins, and circulating histones, play a crucial role in DIC development.^65^ DAMPs can directly influence the coagulation process. NETs facilitate clot formation by providing a surface for blood cells and procoagulant factors to interact, thereby accelerating thrombin production and fibrin clotting. The interaction between NETs and platelets is reciprocal; platelets can activate neutrophils, and NET components, in turn, activate platelets. In thrombus formation, platelets are recruited to NETs through interactions involving C3b deposits and histones, serving as a scaffold for thrombus formation. NETs can also carry neutrophil‐derived TF into the extracellular space and activate factor XII, enhancing thrombin generation through both the intrinsic and extrinsic coagulation pathways. Moreover, NETs, composed of DNA, histones, and granular proteins released from activated neutrophils, can mediate inflammatory responses and lead to microvascular thrombosis and tissue ischemia.^66^

HMGB1, a nonhistone nuclear protein, plays a significant role in coagulation, particularly in platelet activation. In HMGB1‐deficient platelets, bleeding time is prolonged, and platelet aggregation, thrombus formation, inflammation, and organ damage are reduced during experimental trauma/hemorrhagic shock.^67^ HMGB1 is also able to activates the process of NETosis and triggers the expression of TF in monocytes.^68^ In sepsis, HMGB1 levels in the bloodstream correlate with DIC scores and the sequential organ failure assessment, which are crucial for tracking sepsis severity in the ICU.^69^

S100A9, a member of the S100 family, forms calprotectin with S100A8. This heterodimer regulates myeloid function and modulates intracellular calcium signaling, enhancing platelet thrombogenicity by inducing phosphatidylserine exposure and microparticle release.^70^ Activated neutrophils transfer calprotectin to platelets, which increases platelet adhesiveness and aggregation.^71^ A recent study found that S100A8/S100A9, by interacting with the GPIbα receptor on platelets, induced the expression of P‐selectin, activated GPIIb/IIIa, and promoted the release of microvesicles from platelets to form procoagulant platelets.^72^ A recent study found that S100A8/S100A9, by interacting with the GPIbα receptor on platelets, induced the expression of P‐selectin, activated GPIIb/IIIa, and promoted the release of microvesicles from platelets to form procoagulant platelets. Calprotectin binding to glycoprotein 1b on platelets can potentiates VWF‐mediated aggregation, underscoring its role in coagulation.

#### 4.2.2 Pyroptosis and DIC

Pyroptosis, an inflammatory form of cell death, is characterized by the release of inflammatory mediators upon cell demise, thereby triggering an inflammatory response. This process is a crucial component of the innate immune system and significantly contributes to the activation of coagulation.^46^ When caspase‐11 detects intracellular bacterial LPS, it cleaves gasdermin D (GSDMD) into fragments that form nano‐pores in the cell membrane, leading to pyroptosis. The formation of GSDMD pores initiates a systemic coagulation response; the calcium influx through these pores activates transmembrane protein 16F (TMEM16F), a scramblase that promotes the exposure of phosphatidylserine on the cell surface.^73^ This exposure significantly boosts the pro‐coagulant activity of TF, thereby activating the coagulation cascades.^50^

#### 4.2.3 Complement system and DIC

The complement system is closely intertwined with coagulation pathways, amplifying both inflammatory and thrombotic responses. Components such as C3a and C5a, generated during complement activation, can stimulate the assembly of prothrombinase, leading to thrombin generation.^74^, ^75^ Additionally, complement activation upregulates the expression of adhesion molecules on endothelial cells, promoting the attachment and activation of leukocytes, which further contributes to a procoagulant state.^76^ Complement activation products also interact with platelets, enhancing their activation and aggregation, and thus playing a role in thrombus formation.

The term “immunothrombosis” describes the innate intravascular immune response that triggers thrombin production and microthrombi formation.^77^ Initially, immunothrombosis creates an intravascular scaffold that aids in pathogen recognition and eradication, improving endothelial integrity.^78^ However, uncontrolled immunothrombosis can cause tissue damage and contribute to organ dysfunction. The interaction of these elements with the coagulation system can result in a hypercoagulable state, exacerbating the clinical manifestations of DIC.^79^ As a result, PAMPs, DAMPs, NETs, activated immune cells, endothelial cells, and damaged host cells propagate prothrombotic and proinflammatory responses and coagulopathies. Therefore, there must be many regulatory mechanisms in normal human tissues that control this central aspect of the coagulation response.

### 4.3 Amplification of coagulant activity

During the procoagulant state of DIC, natural anticoagulant mechanisms are suppressed.^80^ Endothelial dysfunction, consumption and liver synthesis disorders lead to decreased levels of AT, thrombomulin, protein C (PC), protein S (PS) and TFPI levels. The reductions in anticoagulant factors, in turn, exacerbate anticoagulant activity. Restoration of the function of the anticoagulant molecules is a promising treatment in DIC.

#### 4.3.1 Antithrombin

AT stands as the principal endogenous coagulant inhibitor, a 58‐kDa plasma glycoprotein synthesized by the liver and endothelial cells, and a member of the serine protease inhibitor family.^81^ AT inhibits IIa, Xa, and IXa, and also has a mild inhibitory effect on factors XIa and VIIa. It can suppress platelet aggregation and attachment and possesses anti‐inflammatory properties by reducing cytokine production by neutrophils and endothelium, preventing neutrophil rolling and adhesion, and decreasing interactions between neutrophils and endothelium.^82^ Acquired AT deficiency is common in DIC, caused by ongoing thrombin generation, degradation by enzymes released from neutrophils, loss from circulation due to capillary leakage, and impaired synthesis from liver failure.^83^ In sepsis‐related DIC, decreased AT levels were associated with poor prognosis.^84^ AT is necessary for effective anticoagulation with heparin or low‐molecular‐weight heparin (LMWH), and insufficient AT levels in sepsis patients may reduce the effectiveness of heparin.^85^ In trauma patients, AT deficiency is linked to higher injury severity, hemorrhage, mortality, and fewer ventilator‐free days, indicating its potential in risk assessment.^86^ This highlights the importance of monitoring and managing AT levels for effective treatment strategies.

#### 4.3.2 Thrombomodulin

Thrombomodulin is a key endothelial cell surface glycoprotein that plays a significant role in the regulation of the coagulation system.^87^ It forms a complex with thrombin, thereby inhibiting thrombin's pro‐coagulant activity and promoting the activation of PC, an important natural anticoagulant.^88^ The thrombomodulin–thrombin complex accelerates the conversion of PC to its activated form, APC, which possesses anticoagulant, anti‐inflammatory, and profibrinolytic properties.^89^ In conditions like sepsis and systemic inflammation, the expression of thrombomodulin in endothelial cells is reduced, and its functionality is compromised.

#### 4.3.3 Activated protein C

APC and PS are essential anticoagulant molecules. PC is a double‐chain glycoprotein synthesized by liver megakaryocytes and endothelial cells and is a vitamin K‐dependent factor.^90^ PC can bind to the endothelial PC receptor on endothelial cells, while thrombin also binds to the thrombin receptor.^87^ PC forms a 1:1 complex with thrombin, which leads to the cleavage and activation of PC into APC. On phospholipid surfaces, APC inhibits the coagulation pathway by specifically cleaving the peptide bonds of factors VIIIa and Va with its cofactor, PS, achieving an anticoagulant effect. The primary function of PS is to act as a cofactor for PC, enhancing its inactivation effects.^91^ PC can also lead to a decrease in levels of PAI‐1 and thrombin‐activatable fibrinolysis inhibitor (TAFI), promoting fibrinolysis.^92^ A substantial reduction in the PC system can severely disrupt the proper regulation of activated coagulation. In addition to its anticoagulant functions, APC can also mediate anti‐inflammatory effects and increase endothelial barrier function.^93^, ^94^, ^95^ Clinical trials, such as the PROWESS trial, have demonstrated the benefits of APC in treating sepsis by leveraging both its anticoagulant and anti‐inflammatory properties, although the use has been refined over time to target specific patient populations based on ongoing research and clinical experience.^96^, ^97^

#### 4.3.4 TF pathway inhibitor

TFPI is a plasma serine protease inhibitor synthesized by endothelial cells and is an important anticoagulant substance.^98^ TFPI primarily functions by inhibiting the TF–VIIa complex formation, preventing its activity and thus blocking the initiation of the extrinsic coagulation pathway. Additionally, TFPI can bind to Xa, forming an Xa complex, which then combines with the TF–VIIa complex to form a quaternary complex, exerting its anticoagulant effect. Administering recombinant TF pathway inhibitor (rTFPI) to hinders the formation of blood clots and fibrin accumulation, alleviates fatality rates from septic shock, and guards against the onset of DIC.^99^ Studies have detected high levels of TFPI in individuals with sepsis‐induced DIC, which coincide with high levels of TF, indicating an insufficiency of TFPI to counteract the TF‐triggered coagulation process.

### 4.4 Altered fibrinolytic system

The fibrinolytic system plays a crucial role in the pathogenesis of DIC, representing an ongoing physiological mechanism for the dissolution of fibrin clots. This system maintains the equilibrium between achieving hemostasis following vascular injury and ensuring uninterrupted blood flow to vital organs. Plasminogen, a zymogen synthesized by the liver, circulates in plasma and, upon activation by tissue‐type PA (tPA) or urokinase‐type PA (uPA), is converted into plasmin. Plasmin degrades the fibrin mesh structure, forming soluble fibrin degradation products (FDPs), thereby dissolving blood clots.^100^

The fibrinolytic system, while regulating thrombus formation, is also controlled by various inhibitors. PAI‐1, synthesized by endothelium and megakaryocytes and stored in platelets, regulates fibrinolysis by inhibiting the activity of tPA and uPA. α2‐Antiplasmin prevents excessive fibrinolysis by directly inhibiting plasmin activity.^101^ TAFI, synthesized in the liver and activated by thrombin with thrombomodulin, functions by cleaving lysine residues from fibrin to inhibit plasmin formation and fibrin degradation.^102^

Following major trauma, alterations occur in the coagulation and fibrinolytic systems. Endothelial cells release tPA to initiate fibrinolysis, while PAI‐1 levels remain unchanged. Imbalances of tPA and PAI‐1 induce a hyper‐fibrinolytic phenotype in trauma patients during the initial hours. This phase of enhanced fibrinolysis is short‐lived, typically ending a few hours after PAI‐1 secretion by endothelial cells and, in some cases, platelets. This rapid transition is referred to as “fibrinolytic shutdown.”^103^ In sepsis, fibrinolysis is characterized by heightened coagulation and fibrin production, altered clot structure that is less prone to lysis, and reduced fibrinolysis due to elevated PAI‐1, leading to microthrombi and organ failure.^104^ PAI‐1 is associated with the incidence of DIC and can be a prognostic indicator for sepsis.^105^, ^106^ In acute promyelocytic leukemia (APL), reduced expression of PAI‐1 promote the hyper‐fibrinolytic state.^107^, ^108^ Numerous tumors can exhibit plasminogen‐activating factors such as uPA and tPA, potentially leading to hyperfibrinolysis.^109^ In contrast, some cancers produce PAI‐1, which counteracts fibrinolysis.^48^

In conclusion, fibrinolysis may exhibit reduced (hypofibrinolysis) or increased (hyperfibrinolysis) activity, depending on the pathophysiological characteristics of the underlying disease. The treatment should be cautious to restore the impaired fibrinolysis.

### 4.5 Endothelial cells—throughout the entire coagulation process

Endothelial cells play a pivotal role in coagulation and fibrinolysis.^110^ As the primary producers and repositories of vWF, they facilitate platelet adhesion at sites of vascular injury by interacting with the glycoprotein Ib receptor on platelets. However, during the procoagulant phase of DIC, endothelial cells become damaged or activated. This injury results in the loss of glycocalyx and surface proteins, thereby reducing anticoagulant activity.^111^ The activated endothelium exposes TF, which promotes coagulation, and have decreased thrombomodulin, thus reducing PC activation. The endothelial surface also hosts TFPI, and a reduction in its levels can enhance thrombin production. Vascular damage can lead to a decrease in endothelial TFPI, as observed in thrombotic microangiopathy (TMA) patients with reduced TFPI levels. Imbalances in TF and thrombomodulin on endothelial cells can increase thrombin formation, converting fibrinogen to fibrin and activating platelets. Endothelial cells also produce tPA and uPA, regulating fibrinolysis through PAI‐1. Disruption of the endothelium can decrease fibrinolysis and fibrin clearance. Elevated PAI‐1 levels are associated with a higher thrombotic risk. Endothelial injury leads to reduced fibrinolytic activity and increased PAI‐1, creating an antifibrinolytic environment.^112^ Furthermore, various adhesion molecules expressed on the endothelial surface modulate the binding and activation of leukocytes to the vessel wall, triggering the release of multiple cytokines that can further mediate coagulation activation and suppression of endogenous fibrinolysis.

## 5 CLINICAL MANIFESTATIONS OF DIC

Patients with DIC can experience bleeding and thrombosis, either independently or simultaneously, depending on the phase of DIC they are in. The systemic activation of the coagulation system in DIC can result in consumptive coagulopathy, which manifests as bleeding at various sites. This includes dermatologic bleeding, encephalorrhagia, gastrointestinal bleeding, airway bleeding, genitourinary tract bleeding, and bleeding from surgical sites. These bleeding episodes can lead to persistent hypotension, shock, and organ dysfunction. Thrombosis in DIC may be less apparent and often subclinical. For instance, pulmonary thrombosis can impair gas exchange and damage the alveolar–capillary barrier, leading to hypoxemia and acute respiratory distress syndrome. Microthrombi in the glomeruli and renal tubules can also decrease perfusion, resulting in acute kidney injury. Distinguishing whether organ failure is due to the underlying condition or to microvascular clot formation can be challenging, complicating diagnosis and potentially leading to delays.

The clinical features of DIC are related to its various causes. Sepsis, a primary cause of DIC, often presents as a thrombotic type of DIC with organ dysfunction. In contrast, trauma‐related DIC is characterized by an early fibrinolytic phenotype followed by a subsequent thrombotic response. This difference underscores the need for vigilant monitoring and tailored management strategies in different patient populations.

## 6 DIAGNOSIS

Considering the diverse pathogenic factors and complex mechanisms involved in DIC, definitive diagnosis of DIC cannot be achieved with a single clinical test or examination index. Current DIC diagnostic algorithms mainly utilize a combination of conventional coagulation indicators to facilitate ease of use and rapid detection of DIC (Table 2). The main DIC scoring systems, which are continuously updated, have been established by the ISTH, JAAM, JMHLW, and JSTH.^113^, ^114^ In China, the Thrombosis and Hemostasis Group of the Hematology Branch of the Chinese Medical Association has developed the Chinese Disseminated Intravascular Coagulation Scoring System through multicenter, large‐sample retrospective and prospective studies.^115^

**TABLE 2:** Diagnostic criteria for DIC.

Measurement	Score	ISTH overt	JAAM	SIC
Platelet (10^9^/L)	0	>100	≥120	≥150
	1	≤100	≥80, <120	<150
	2	<50		<100
	3		<80	
PT (s)	0	<3	<1.2 (PT ratio)	
	1	≥3, <6	≥1.2	≥1.2, < 1.4
	2	≥6		INR > 1.4
FDPs (mg/L)	0	DDI < 1	FDP < 10	
	1		10 ≤ FDP < 25	
	2	1 ≤ DDI < 5		
	3	DDI ≥ 5	≥25	
Fg (g/L)	0	>1.0	>3.5	
	1	≤1.0	≤3.5	
SIRS score	0–2		0	
	≥3		1	
Organ dysfunction	1			SOFA = 1
	2			SOFA ≥ 2
DIC score		DIC ≥ 5	DIC ≥ 5	≥4

Abbreviations: FDPs, fibrin degradation products; PT, prothrombin time; SIRS, systemic inflammatory response syndrome.

The ISTH first defined overt DIC in 2001 with criteria that include thrombocytopenia, significant prolongation of prothrombin time (PT), moderate to high levels of fibrin‐related markers, and reduced fibrinogen levels. These markers suggest severe, widespread activation of the coagulation system, resulting in both clot formation and breakdown.^116^ The overt DIC scoring system is widely being applied and has adequately precise for diagnosing DIC in ICU patients.^6^ However, a primary limitation of this scoring system is that by the time overt DIC is detected, the condition may have advanced to a point where treatment responses are suboptimal. Therefore, identifying nonovert DIC and the early stages of DIC remains a significant challenge and a critical area for improvement in DIC diagnosis and management.

The initial ISTH scoring system for nonovert DIC incorporates a kinetic component that compares results from two consecutive measurements. A deterioration in the parameters raises the score, whereas an improvement lowers it. This method enhances the sensitivity of the parameters but necessitates daily measurements, which may not be practical in all clinical settings. Moreover, studies have not yet established that the nonovert DIC score can reliably distinguish between nonovert DIC and overt DIC.

In light of these limitations, especially regarding early detection and specific contexts such as sepsis, the ISTH introduced the sepsis‐induced coagulopathy (SIC) criteria in 2017. These criteria were designed to address the shortcomings of existing criteria and provide a more accurate tool for the early identification of DIC in septic patients.^117^ SIC includes a SOFA (sequential organ failure assessment) score greater than 2 points, platelet count, and PT, thus it is easy to calculate clinically. The total score helps to categorize the degree of coagulation dysfunction and determine the appropriate clinical management. The SIC criteria is increasingly adopted in clinical practice. Studies have showed that SIC is associated with higher incidence and mortality rates and could be interpreted as an early warning signal for impending sepsis‐related damage.^9^, ^118^, ^119^ The European Society of Cardiology and ISTH's joint consensus statement on antithrombotic therapy in severe infections references SIC for coagulopathy diagnosis and advocates SIC score‐guided therapy.^120^ The SIC score shows potential in identifying high‐risk patients for early DIC development.

The JAAM criteria was also introduced to provide a practical and clinically applicable approach for diagnosing nonovert DIC, particularly in patients with acute conditions such as sepsis.^7^ These include decreased levels of AT, PC, and increased thrombin–AT (TAT) complexes. However, the molecular markers used for diagnosing nonovert DIC are not widely implemented due to their complexity and cost. The JAAM DIC criteria have been proved to be outdated, particularly after the update of the sepsis definition to Sepsis‐3. In response, the JAAM DIC score was recently modified to align with the new definition of sepsis, JAAM‐2 DIC criteria, replacing the systemic inflammatory response syndrome (SIRS) criterion.^121^ The alignment of this set of criteria with the SIC guidelines put forth by the ISTH remains to be confirmed.

### 6.1 Potential diagnostic markers

Current DIC score algorithms provided an venue for simple and rapid diagnosis for DIC. When overt DIC is detectable, it may have progressed to an irreversible state, making it too late for effective treatment. Attempts has been made to detect DIC in its early stages (nonovert, compensatory) before it reaches an irreversible phase. Thrombotic and fibrinolytic phenotype have distinct molecular process. DIC diagnosis combined with molecular markers could be more sensitive for monitoring the coagulation/fibrinolysis state. Biomarkers indicative of thrombin generation (such as TAT, prothrombin fragment 1 + 2, ex vivo TG assay),^122^, ^123^ fibrinolysis activity (plasminogen, PAP complex, tPA, α2‐antiplasmin, PAI‐1, TAFI, plasma‐based fibrin formation and lysis assays) are utilized to evaluate the DIC phenotype, along with its severity and progression.^124^, ^125^ By using new models incorporating these markers, more specific and adequate DIC management could become possible. In addition, DIC is a complex process involving platelets, coagulation factors, endothelium, and the immune system. The assessment of DAMPs, such as HMGB1, netosis, nucleosomes, extracellular histones, and cell‐free DNA, in combination with other indicators might offer supplementary insights for diagnosis in sepsis‐associated DIC.^126^ Platelet function analysis such as soluble P‐selectin, Beta‐thromboglobulin, soluble glycoprotein, platelet aggregation test, platelet adhesion index is also under investigation.^60^ Platelet‐derived procoagulant microparticles play a significant role in DIC, and these platelet‐derived microparticles could become a new biomarker for DIC. Recently, microthrombi, which are considered to be amyloid‐fibrin(ogen) aggregates, are shown to be associated with a variety of conditions, and in sepsis, SARS‐CoV‐2 infection. Studies indicate that microthrombi in critically ill patients are correlated with the diagnosis of sepsis, and can predict sepsis‐related coagulopathy and adverse clinical outcomes.^127^ The diagnostic and prognostic value of these biomarkers remains to be established in large‐scale studies. As the aforementioned research progresses, the understanding of the detailed pathophysiological differences between underlying diseases continues to advance, and disease‐specific diagnostic criteria for DIC are very important for future development.

### 6.2 Point‐of‐care tests

While the JAAM DIC criteria and SIC are effective for identifying thrombotic DIC, they are less suitable for detecting early bleeding, or the enhanced fibrinolysis type of DIC, which is often seen in trauma and obstetric emergencies. Beyond traditional coagulation indicators, there is an ongoing effort to quickly identify early‐stage DIC to prevent its progression to a severe and potentially irreversible state.^126^ Viscoelastic tests such as thromboelastography (TEG) or rotational thromboelastometry (ROTEM) present an alternative laboratory approach for the rapid diagnosis and prognosis of DIC.^128^ Traditional coagulation tests require up to 40–60 min for results while viscoelastic tests offer real‐time data, aiding in the swift identification of patients at high risk for severe bleeding. These tests assess not only coagulation time but also velocity, clot firmness, and lysis index, offering more comprehensive information about clot formation capabilities than APTT and PT.^129^ However, the efficacy of standard viscoelastic tests in detecting early procoagulant activity before overt DIC is not yet conclusively proven. Conversely, a reduction in clot formation ability as assessed by TEG/ROTEM is associated with overt DIC, as defined by the ISTH DIC scoring, and is correlated with higher mortality rates in several sepsis cohorts.^130^ Moreover, viscoelastic tests are sensitive to overt hyperfibrinolysis, which can guide treatment decisions for DIC patients with bleeding who may be candidates for antifibrinolytic drugs, such as those with trauma‐induced coagulopathy.^131^

### 6.3 Advancements in DIC screening technologies

Recent advancements in nanotechnology and point‐of‐care devices have introduced innovative methods for diagnosing and screening DIC. Advanced microfluidic systems are now capable of mimicking blood coagulation processes under physiological conditions, facilitating molecular‐level assessments of coagulation events.^132^ These devices can rapidly measure various parameters—electrochemical, optical, and mechanical—providing coagulation test results within minutes, which is essential for urgent diagnostic and therapeutic interventions.^133^ Nanomaterials, including gold nanoparticles and graphene oxide, have demonstrated enhanced sensitivity and specificity in DIC diagnostics due to their unique physicochemical characteristics.^134^ Photoacoustic imaging, which combines the deep penetration of acoustic waves with the high contrast of optical imaging, has been used for real‐time monitoring of circulating clots and blood coagulation in tissue.^135^ This technology has shown promise in the detection of heparin and LMWH, with a significant and dose‐dependent increase in photoacoustic signal in the presence of these anticoagulants.^136^, ^137^ Electrochemical sensors, particularly those based on aptamers, have been developed for the detection of thrombin, a key enzyme in the coagulation cascade. These sensors offer rapid, selective, and sensitive detection of thrombin, which is crucial for the diagnosis of DIC.^138^ Fluorescent probes have been used to track clot molecules and cells, allowing for the quantitative measurement of multiple targets simultaneously. This enhances the visualization of clot dynamics and may facilitate noninvasive imaging of early‐stage thrombosis in clinical settings.^139^

Notably, the progress in AI and machine learning has led to the development of algorithms that can monitor various parameters concurrently, enabling the identification of unique patterns in patients. This capability is instrumental in determining the most appropriate treatment or diagnosing, especially for patients with unique conditions, including those undergoing newer anticoagulation therapies.^140^, ^141^

Overall, for suspected DIC cases, it is recommended to use a validated scoring system integrating various lab tests for reference. Since DIC can fluctuate between different phenotypes, parameter changes should be monitored regularly. Underlying DIC‐associated causes should be considered for accurate predictive value, other coagulation disruptors should also be considered to avoid miscalculating the DIC score. New technologies in the diagnosis and treatment of DIC offer significant advancements, potentially revolutionizing patient care. These innovations hold significant promise for more accurate, efficient, and personalized DIC management.

## 7 DIFFERENTIAL DIAGNOSIS OF DIC AND TMA

TMA, manifests as a clinical syndrome characterized by microangiopathic hemolytic anemia, is increasingly gaining the attention of clinicians. TMA encompasses hemolytic anemia, thrombocytopenia, and organ dysfunction, particularly affecting the kidneys and central nervous system, as well as other organs.^142^ Thrombocytopenia and potential organ dysfunction are common clinical features of DIC and TMA, thus differential diagnosis between DIC and TMA is important (Table 3). The core pathogenesis of TMA lies in the abnormal activation and consumption of platelets, as well as endothelial cell dysfunction, while coagulation and fibrinolytic system were not activated in most circumstances.^143^ TMA is more commonly associated with concurrent conditions such as infections, pregnancy, autoimmune diseases, or malignant hypertension. Thrombotic thrombocytopenic purpura (TTP) and hemolytic uremic syndrome (HUS) are used to be considered as the primary causes of TMA syndromes. Unlike DIC that has no specific markers, TMA has some diagnostic markers. TTP requires a markedly decreased ADAMTS13 level, that of STEC–HUS requires the detection of a STEC infection. aHUS involves identifying abnormalities in the complement system.^144^ Concerning treatment, platelet transfusion is contraindicated for TMA, whereas is advised for DIC with thrombocytopenia and major bleeding. Antifibrinolytic therapy is suggested for DIC patients with hyperfibrinolysis. Plasma exchange is recommended for certain TMA cases like TTP but not for DIC. AT concentrate and recombinant thrombomodulin are commonly used for DIC, while eculizumab is effective for complement‐mediated TMA, such as aHUS, and rituximab is beneficial for TTP in patients with high ADAMTS13 inhibitor titers.

**TABLE 3:** Differential diagnosis of DIC and TMA.

	DIC	TMA
Clinical manifestations		
Thrombocytopenia	Frequent	Frequent
Bleeding and bleeding tendency	Frequent	Frequent
Anemia	Often	Usually
Shock or micro circulation dysfunction	Often	Rare
Organ dysfunction	Lung, kidney, shock	Kidney, CNS
Hematuria	Sometimes	Frequent
Pathological mechanisms		
Microvascular thrombosis	Mostly in venules	Mostly in arterioles
Platelet activation	Yes	Yes
Endothelial dysfunction	Endothelial injury	Edema of endothelial cells and subendothelial spaces, accompanied by thickening of vascular walls and platelet microthrombus
Activation of coagulation system	Yes	No
Fibrinolysis	Secondary	No
Laboratory data		
Platelet count	Low	Low
Hemoglobin	Often low	Low
Fibrin related markers	Significantly high	Slightly high
Prothrombin time	Prolong	Normal
Antithrombin	Low	Normal
Albumin	Low	Normal
Creatinine	Often high	High
Total bilirubin, LDH	Often high	High
Treatment	Recommendation: Supportive therapy, blood transfusion (RBC, FFP, PC), anticoagulant, AT, rhTM, etc.	Recommendation: Supportive therapy, blood transfusion (RBC, FFP), PE/FFP, hemodialysis, MABs (on condition), etc.

Abbreviations: AT, antithrombin; DIC, disseminated intravascular coagulation; FFP, fresh frozen plasma; MABs, monoclonal antibodies.; PC, platelet concentrate; PE, plasma exchange; RBC, red blood cell; rhTM, recombinant human thrombomodulin; TMA, thrombotic microangiopathy.

## 8 TREATMENT

**FIGURE 4:**
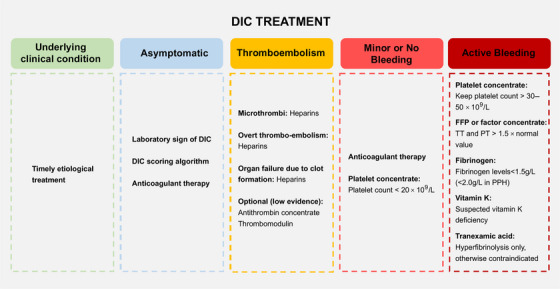
The causes and treatments of DIC. Treatment is divided into addressing the underlying condition and specific management for asymptomatic patients, thromboembolism, and bleeding, with recommendations for LMWH prophylaxis, antithrombin, platelet concentrates, and other supportive therapies based on clinical presentation.

Given the intricate pathophysiology of DIC, its treatment is a multifaceted challenge that demands a personalized approach tailored to the clinical context and the specific underlying cause. In the case of nonovert DIC, addressing the root cause is crucial, as effectively managing the underlying issue can often lead to the resolution of the coagulopathy. However, as DIC advances, initiating effective anticoagulation therapy during the hypercoagulable phase becomes necessary. Despite this, determining the optimal timing for starting anticoagulation and assessing the potential risks, such as bleeding, remain significant challenges in the field (Figure 4).

The use of blood products and clotting factor concentrates may be necessary to manage bleeding resulting from the consumption of platelets and coagulative factors. In essence, the overarching goal is to restore the pathological coagulopathy to a physiological hemostatic state, a concept that is currently the subject of extensive research.

### 8.1 Basic treatment

Treatment of the underlying disorder is the most important principle in DIC management. For instance, DIC due to sepsis or septic shock should focus on the basic tenets of sepsis care, which includes early identification and treatment of the infection, hemodynamic support, and management of organ dysfunction. In trauma, this may involve surgery to control bleeding and repair damaged tissues. Timely etiological treatment helps to reverse and even repair the DIC process.

### 8.2 Anticoagulant treatment

Anticoagulant therapy is recommended when the coagulation system activation and systemic thrombin production overwhelms fibrinolysis and consumption coagulopathy. In sepsis, procoagulant activity and fibrinolytic suppression lead to thrombin formation and organ dysfunction, where anticoagulant treatment is recommended. DIC in some solid cancers is mainly characterized by procoagulant activity and fibrin deposition, appropriate anticoagulant treatment is also suggested. While there is no definitive guideline on the optimal duration for anticoagulant treatment, evidence indicates that initiating therapy promptly, ideally within 24 h, is crucial to capitalize on therapeutic windows in sepsis‐induced DIC.^145^, ^146^ Notably, in cases of DIC induced by severe trauma and traumatic shock, accompanied by critical bleeding attributed to both consumption coagulopathy and excessive fibrinolysis, anticoagulants are considered contraindicated.

#### 8.2.1 AT treatment

As previously mentioned, AT is a serine protease inhibitor that plays a crucial role in the natural anticoagulation system by neutralizing thrombin and other coagulation factors. In DIC, where there is an overwhelming activation of coagulation pathways, AT levels can be significantly depleted, leading to a hypercoagulable state.^147^ In sepsis, where AT activity is markedly suppressed, the use of AT concentrate has been extensively studied, with a significant reduction in 28‐day mortality observed in patients with sepsis‐associated DIC treated with AT. The benefits were particularly pronounced when AT was not co‐administered with heparin.^148^, ^149^ However, a meta‐analysis did not find a significant reduction in overall mortality with AT treatment across all critically ill patients, and subgroup analyses also suggested no potential benefits in patients with DIC and sepsis.^150^ Additionally, AT treatment appears to increase the risk of bleeding, as noted in a Cochrane review that included 30 RCTs; however, the concurrent use of heparin should be considered a risk factor.^151^ The Japanese sepsis guidelines recommend the use of AT for sepsis‐associated DIC, supported by a propensity score‐matched analysis showing improved survival rates.^152^, ^153^ The rationale for this recommendation is based on the understanding that severe insults leading to systemic thrombin generation can overwhelm the body's natural anticoagulant mechanisms, including AT. Restoring these pathways with AT concentrate is thought to mitigate the hypercoagulable state and reduce the associated mortality. Recent evidence on the efficacy and safety of AT has become more favorable in sepsis and sepsis‐associated DIC, although optimal dosing and patient selection for AT therapy remain contentious issues that require further research.^154^, ^155^ AT administration could also be considered in acquired AT deficiency such as DIC‐associated trauma, burns, complicated pregnancy.

#### 8.2.2 Heparin treatment

In DIC cases where thrombosis is the predominant clinical feature, the ISTH recommends the use of unfractionated heparin or LMWH. However, no randomized controlled trials (RCTs) have yet demonstrated a clinically significant outcome for DIC patients. A meta‐analysis suggested potential benefits of unfractionated heparin in reducing mortality in sepsis, particularly in patients with high severity.^156^ The application of heparin in sepsis‐associated DIC is complex, as it may influence not only coagulation pathways but also inflammatory processes, which are pivotal to sepsis pathophysiology.^157^, ^158^ A recent study showed that heparin could prevent caspase‐11‐dependent immune responses and sepsis lethality, independent of its anticoagulant effects.^159^

In a randomized controlled study aiming at evaluating effect of LMWH in COVID‐19 patients at risk of thrombosis (plasma D‐dimer level greater than four times the upper limit or SIC score ≥4), therapeutic doses of LMWH reduced major thromboembolism and death compared with institutional standard heparin thromboprophylaxis, while the treatment effect was not observed in critically ill patients.^160^ Heparins may be most effective when administered early in the disease course to prevent both macrovessel and microvascular thrombosis in this condition. Prophylactic use of heparin or LMWH is advocated in critically ill, nonbleeding DIC patients to prevent venous thromboembolism.

Heparin use should be approached with caution or suspended in DIC patients experiencing bleeding, those at high risk of bleeding, or when platelet counts drop below 20 × 10^9^/L.^161^ For patients with APL‐related DIC, heparin use requires careful consideration, with platelet counts ideally above 20 × 10^9^/L and bleeding risk factored into decisions. In obstetric DIC, which mainly presents with bleeding, the use of unfractionated heparin or LMWH is uncertain and should be reserved for situations where thrombosis is a more immediate concern.

Heparin‐induced thrombocytopenia (HIT), marked by a decrease in platelet count and an increased risk of venous or arterial thrombosis, is a severe complication of heparin therapy.^162^ HIT is caused by synthesis of antibodies targeting platelet factor 4 (PF4) modified by heparin.^163^ When HIT is suspected, heparin therapy should be discontinued immediately, a Doppler ultrasound of the lower limbs conducted, and an alternative anticoagulant such as danaparoid sodium or argatroban prescribed.^164^

### 8.3 Other anticoagulant proteins

During the hypercoagulable phase of DIC, major anticoagulant substances, including APC, TFPI, and the endothelial thrombomodulin, are suppressed. Therefore, drugs that can restore the impaired anticoagulant pathways are also under investigation. Here is an introduction of the current clinical research about main anticoagulant substances: APC, TFPI, and thrombomodulin. It is worth noting that the clinical application potential of these anticoagulant substances is still under investigation and requires further validation.

#### 8.3.1 Recombinant human soluble thrombomodulin

In DIC, where the equilibrium between coagulation and anticoagulation is disrupted, thrombomodulin has emerged as a promising therapeutic option. Clinical trials, particularly in Japan, have investigated the use of recombinant human soluble thrombomodulin (rTM) in patients with DIC stemming from hematologic malignancies or severe infections, showing potential benefits.^165^ A meta‐analysis encompassing 1409 patients from three RCTs and nine observational studies revealed a reduced risk of death in sepsis‐induced DIC patients treated with rTM, suggesting better outcomes.^166^ However, the SCARLET study, a RCT investigated the effect of rTM on 28‐day mortality in patients with SIC showed no risk reduction in the intervention group compared with the placebo group, while the risk of major bleeding is increasing.^167^ Yet, a post‐hoc analysis of the SCARLET study indicated that the mortality risk reduction was most pronounced in subgroups of patients with increased levels of TAT and prothrombin fragment suggesting that rTM may be particularly effective in patients with higher disease severity.^168^ A following updated meta‐analysis found statistically significant difference in mortality risk reduction in SIC without increasing the bleeding risk.^169^ Though the results differ to some extent, overall, the data support the positive effects of using rhTM for the treatment of DIC. Ongoing research aims to clarify the ideal role of rTM in treating DIC and DIC‐associated causes.

#### 8.3.2 Recombinant APC

Recombinant APC (rAPC) was the first natural anticoagulant to be approved for the treatment of sepsis, following the results of the PROWESS trial, which showed a beneficial effect in patients with severe sepsis.^170^ A subgroup analysis of the PROWESS trial indicated an even more beneficial effect on survival in patients with overt DIC. However, subsequent trials failed to demonstrate a consistent benefit of rAPC, and it was associated with an increased risk of bleeding, leading to its withdrawal from the market.^171^, ^172^ The withdrawal of rAPC from the market highlights the complexity and challenges in treating DIC. While rAPC showed promise in certain patient populations, its broader application was limited due to safety concerns.^173^ This underscores the need for a nuanced approach to the use of anticoagulants in DIC, where the risk of bleeding must be carefully balanced against the potential benefits of treatment.

#### 8.3.3 Recombinant TF pathway inhibitor

TFPI is an endogenous inhibitor of the coagulation system that directly inhibits factor Xa and the TF/factor VIIa complex. Given its role in inhibiting the initiation of the coagulation cascade, TFPI theoretically represents a promising target for the treatment of DIC. Early studies, including animal models and trials in healthy individuals, suggested that rTFPI could be a viable treatment option.^174^ However, the transition from theoretical promise to clinical efficacy has been challenging. A phase II trial of rTFPI in patients with severe sepsis reported a nonsignificant reduction in 28‐day mortality.^175^ The subsequent phase 3 trial, known as the OPTIMIST trial, failed to show a survival benefit for patients with severe sepsis receiving rTFPI compared with placebo.^176^ These results highlight the gap between the theoretical mechanisms of action and the clinical outcomes in complex conditions such as DIC.

In conclusion, while thrombomodulin, APC, and TFPI have shown theoretical promise in the treatment of DIC, their clinical efficacy has been variable. Thrombomodulin, particularly in its recombinant form, has demonstrated the most consistent potential benefit, especially in patients with more severe disease manifestations. The challenges faced by rAPC and rTFPI in clinical trials emphasize the need for a more tailored approach to treatment, taking into account the specific characteristics and severity of DIC in individual patients. Ongoing research and clinical trials are essential to refine our understanding of these treatments and to identify the patient populations most likely to benefit from them.

### 8.4 Fibrinolysis restoration

In the context of sepsis, where fibrinolysis is frequently disrupted, there is a significant interest in developing therapeutic agents that target proteins that inhibit fibrinolysis. Particularly, the inhibition of PAI‐1 is seen as valuable due to its strong correlation with poor outcomes.^106^ The development of small‐molecule PAI‐1 antagonists has shown potential in inhibiting thrombus formation and has been deemed safe in animal models.^177^ PAI‐1 inhibitor has the ability to restore clot lysis in plasma and decrease pulmonary microthrombus formation and hemorrhage in a murine sepsis model.^178^ Despite these promising results, clinical trials for PAI‐1 inhibitors are still pending. PAI‐1 inhibitor does not influence thrombus formation or fibrinolysis in a range of established human plasma and whole blood systems.^179^ In a Japanese trial for COVID‐19 pneumonia, PAI‐1 inhibitor showed nonsignificant trends in improved oxygenation and reduced oxygen therapy days compared with placebo, but larger studies are needed to confirm its efficacy.^180^

The use of profibrinolytic therapies like recombinant tissue PA (rtPA) for sepsis‐induced DIC has also been reported.^181^ rtPA treatment can effectively suppress PAI‐1 activity in the LPS‐induced DIC model in rats and improve organ dysfunction.^182^ However, systemic administration of rtPA has been linked to a significant risk of bleeding complications, including intracranial hemorrhages, which poses safety concerns, particularly for DIC patients who are prone to both microthrombi and bleeding. So far, there have been no prospective studies assessing the systemic use of recombinant tPA in the context of sepsis or DIC. Still, with the appropriate selection of targets and the design of optimal administration methods, rtPA may offer a new therapeutic agent for DIC and is worth considering for future study.

### 8.5 Antifibrinolysis treatment

In DIC, the system that regulates fibrinolysis can become overwhelmed or suppressed, depending on the pathophysiology of underlying diseases. In sepsis, fibrinolysis system is commonly impaired, antifibrinolysis treatment is not recommended, unless hyperfibrinolysis is clearly indicated. There is limited evidence regarding their benefits or safety in this context. In the setting of trauma, initial activation of fibrinolysis induced extensive tissue injury and massive bleeding are common, antifibrinolytic agents such as tranexamic acid (TXA) play a crucial role. The CRASH‐2 trial demonstrated that early administration of TXA within 3 h of injury reduced mortality in bleeding trauma patients.^46^ Hyperfibrinolysis significantly contributes to bleeding and coagulopathy in peripartum hemorrhage. Early use of TXA in critically ill postpartum patients with hemorrhage is recommended.^183^ In APL, a condition where both DIC and hyperfibrinolysis coexist, antifibrinolytic therapy may be appropriate. The use of TXA in these scenarios is supported by clinical data, emphasizing the importance of timely intervention.

### 8.6 Substitution therapy in bleeding and overt DIC

Substitution therapy is a critical component in the management of DIC, particularly in patients with active bleeding or at high risk of bleeding complications.^184^, ^185^ This therapy involves the transfusion of platelets, fresh frozen plasma (FFP), and the use of coagulation factor concentrates. The ISTH provides guidance on treatment thresholds for these therapies. Platelet concentrates are recommended for DIC patients experiencing significant bleeding, with a threshold set at 50 × 10^9^/L. For DIC patients with minimal or no bleeding, a lower threshold of 20 × 10^9^/L is generally accepted. Substitution with coagulation factors is advised for patients with major bleeding and significantly prolonged APTT or PT. FFP is the preferred initial treatment. Prothrombin complex concentrate (PCC) is also an alternative, containing vitamin K‐dependent factors but lacking some crucial ones. PCC can be used with caution in actively bleeding patients, but the risk of thromboembolism must be monitored. Vitamin K can help with deficiencies in vitamin K‐dependent factors but takes over 6 h to be effective. For low fibrinogen levels, fibrinogen concentrate or cryoprecipitate is used, aiming to maintain levels above 1.5 g/L, or above 2.0 g/L for postpartum hemorrhage. The role of recombinant human activated factor VII (rhFVIIa) in severe bleeding DIC has also being explored, with no proven effectiveness and potential risks. However, it should be noted that for now no clinical trials has been established to prove the efficacy of such treatment. During the hypercoagulable phase, blood transfusions should be avoided. In the consumption coagulopathy and overwhelmed fibrinolysis induced bleeding, transfusion therapy could be implemented. Correctly assessing the pathological process of DIC before initiating replacement therapy is crucial, during substitution treatment, coagulation indicators should be monitored dynamically to avoid potential risks, such as thrombosis.

### 8.7 Experimental and emerging therapies targeting immunothrombosis

The intricate interaction between the innate immune system and coagulation following infection or injury, a process known as immunothrombosis, is a primary cause of DIC. Identifying new therapies to block the immunothrombotic triggering of TF, which may involve inhibiting pyroptosis to limit TF release or using cysteine modification therapies to directly target TF unmasking, shows potentiality.^73^ The potential of drugs like dimethyl fumarate and 4‐octyl itaconate to suppress TF release by inhibiting IFN and caspase‐11 pathways highlights the promise of this strategy in controlling TF‐mediated coagulopathy.^186^

We agree with the view that the new directions in DIC therapy tend to develop safer and more effective treatment strategies that target not only coagulation pathways but also anti‐inflammatory and cytoprotective mechanisms.^187^ These new therapies aim to reduce the risk of bleeding while providing the benefits of antithrombotic and anti‐inflammatory effects. For example, by targeting adhesion molecules on platelets (P‐selectin, GPIb, αIIbβ3) and neutrophils, the formation of neutrophil‐platelet aggregates can be inhibited, improving microvascular dysfunction and inflammation.^187^ These new therapeutic approaches are still under investigation.

The above content includes the routine treatment procedures for DIC, as well as novel clinical studies that are still under exploration. Overall, etiology identification and management are the cornerstones of DIC. The management of DIC necessitates a delicate balance between treating the coagulopathy and avoiding exacerbation of the underlying condition. For future treatment, the integration of advanced diagnostic tools, such as microfluidic devices, and novel molecular markers, allows for a more personalized approach for therapy. These provide real‐time data on coagulation dynamics, enabling clinicians to make informed decisions about the timing and type of therapeutic interventions. With an evolving understanding of DIC's complex pathophysiology, the development of precise, patient‐specific treatments is becoming more attainable, offering new hope for management. The design of clinical trials for novel therapies presents a promising avenue to not only address the coagulopathy itself but also to target the underlying causes of DIC. The future of DIC treatment lies in the continued exploration of novel therapies, with a focus on individualized patient care and the integration of emerging evidence into clinical practice. This shift toward precision medicine in DIC treatment has the potential to significantly improve patient outcomes and revolutionize the management of this complex and often devastating condition.

## 9 CONCLUSION AND PERSPECTIVE

DIC is a complex syndrome characterized by systemic activation of coagulation, leading to thrombosis and bleeding. Diagnosis remains challenging due to varying etiologies and presentations, prompting the search for novel biomarkers to enhance early detection, especially in sepsis, trauma, and obstetric emergencies where DIC can be elusive. The design of clinical trials for novel therapies is a promising avenue, aiming to address the underlying cause and the coagulopathy itself. Considering the heterogeneity of DIC across different patient populations, future research may unveil more targeted personalized treatment strategies to improve outcome. As our understanding of DIC's pathophysiology deepens, the prospects for precise, patient‐specific treatments become increasingly feasible, offering hope for better management of this multifaceted condition.

## AUTHOR CONTRIBUTIONS

Fangchen Gong, Xiangtao Zheng, and Shanzhi Zhao contributed equally to this work. Fangchen Gong, Xiangtao Zheng, and Shanzhi Zhao were responsible for the conception and design of the review. Huan Liu performed the literature search and data analysis. Erzhen Chen and Rongli Xie contributed to the interpretation of the findings and critically revised the manuscript. Ranran Li and Ying Chen provided overall guidance and supervision, and also contributed to the final revision of the manuscript. All authors have read and approved the final version of the manuscript.

## CONFLICT OF INTEREST STATEMENT

The authors have declared that no conflict of interest exists.

## ETHICS STATEMENT

Not applicable.

## Data Availability

Not applicable.
